# Risk of Guillain-Barré Syndrome Among Older Adults Receiving Influenza Vaccine in Taiwan

**DOI:** 10.1001/jamanetworkopen.2022.32571

**Published:** 2022-09-21

**Authors:** Cheng-Chang Yen, Kai-Che Wei, Wen-Hwa Wang, Yu-Tung Huang, Yu-Chia Chang

**Affiliations:** 1 Division of Neurology, Department of Internal Medicine, Kaohsiung Veterans General Hospital, Kaohsiung, Taiwan; 2 Department of Physical Therapy, Shu-Zen Junior College of Medicine and Management, Kaohsiung, Taiwan; 3 Department of Dermatology, Kaohsiung Veterans General Hospital, Kaohsiung, Taiwan; 4 School of Medicine, College of Medicine, National Yang Ming Chiao Tung University, Taipei, Taiwan; 5 Division of Cardiology, Department of Internal Medicine, Kaohsiung Veterans General Hospital, Kaohsiung, Taiwan; 6 Health Management Center, Kaohsiung Veterans General Hospital, Kaohsiung, Taiwan; 7 College of Management, I-Shou University, Kaohsiung, Taiwan; 8 Center for Big Data Analytics and Statistics, Department of Medical Research & Development, Chang Gung Memorial Hospital, Linkou Main Branch, Taoyuan, Taiwan; 9 Department of Health Care Management, College of Management, Chang Gung University, Taoyuan, Taiwan; 10 Department of Long Term Care, College of Health and Nursing, National Quemoy University, Kinmen County, Taiwan; 11 Department of Healthcare Administration, College of Medical and Health Science, Asia University, Taichung, Taiwan

## Abstract

**Importance:**

Although influenza vaccination has been associated with Guillain-Barré syndrome (GBS), the findings among studies of older adult populations are inconsistent.

**Objective:**

To determine the risk of GBS after influenza vaccination among older adults.

**Design, Setting, and Participants:**

This cross-sectional study incorporated a self-controlled case series design. Days 1 to 7, days 1 to 14, and days 1 to 42 after influenza vaccination were identified as risk intervals; days 8 to 180, days 15 to 180, and days 43 to 180 comprised the corresponding control interval. Population-based data were obtained from Taiwan’s National Health Insurance research database between January 1, 2003, and December 31, 2017. Data were analyzed from November 1, 2021, through February 28, 2022. Adults 65 years or older who developed GBS within 180 days after influenza vaccination were enrolled.

**Exposure:**

Government-funded seasonal influenza vaccination.

**Main Outcomes and Measures:**

Onset of GBS during risk intervals after influenza vaccination compared with control intervals using Poisson regression to calculate incidence rate ratio (IRR).

**Results:**

Of 13 482 122 adults aged 65 years or older who received an influenza vaccination, 374 were hospitalized for GBS. The mean (SD) age of the study population was 75.0 (6.1) years; 215 (57.5%) were men and 159 (42.5%) were women. In terms of comorbidities, 33 adults (8.8%) had cancer and 4 (1.1%) had autoimmune diseases. The IRRs for GBS during days 1 to 7, days 1 to 14, and days 1 to 42 were 0.95 (95% CI, 0.55-1.61; *P* = .84), 0.87 (95% CI, 0.58-1.29; *P* = .48), and 0.92 (95% CI, 0.72-1.17; *P* = .49), respectively. No results showed statistical significance. Similarly, no significant differences in IRRs were noted for the overall risk interval (ie, days 1-42) in subgroup analyses pertaining to different age groups (65-74 years [0.93 (95% CI, 0.66-1.31)], 75-84 years [0.85 (95% CI, 0.58-1.26)], and ≥85 years [1.10 (95% CI, 0.57-2.11)]), sex (men, 0.97 [95% CI, 0.71-1.33; *P* = .87]; women, 0.85 [95% CI, 0.58-1.23; *P* = .39]), Charlson Comorbidity Index (1.03 [95% CI, 0.77-1.38; *P* = .84]), or comorbidities (cancer, 0.68 [95% CI, 0.28-1.64; *P* = .39]; autoimmune disease, 1.10 [95% CI, 0.11-10.53; *P* = .94]).

**Conclusions and Relevance:**

These findings suggest that influenza vaccination did not increase the risk of GBS among adults aged 65 years or older in Taiwan regardless of postvaccination period or underlying characteristics.

## Key Points

**Question:**

Is influenza vaccination associated with Guillain-Barré syndrome (GBS) among adults aged 65 years or older?

**Findings:**

This cross-sectional study of 374 older adults in Taiwan who were hospitalized for GBS found that the risk of GBS did not increase after influenza vaccination regardless of postvaccination risk interval or baseline characteristics.

**Meaning:**

These findings suggest that influenza vaccination may not increase the risk of GBS among older adults.

## Teaser

This cross-sectional study investigates the association between seasonal influenza vaccination and incidence of Guillain-Barré syndrome among adults 65 years or older in Taiwan.

## Introduction

Guillain-Barré syndrome (GBS) is a rare and severe disorder in which the immune system attacks the peripheral nerves.^1,2,3^ GBS is characterized by muscle weakness and paralysis that progress rapidly but rarely lead to death.^1,3^ The syndrome is usually caused by an antecedent infection that results in an aberrant autoimmune response targeting peripheral nerves and their spinal roots.^3,4^ Infections of the upper respiratory tract and the gastrointestinal tract are the most common antecedent infections. An interval of 1 to 4 weeks is commonly observed between infection and onset of neurological illness.^5^ The estimated incidence rate ranged from 0.81 to 1.89 cases per 100 000 person-years according to many studies that have been conducted in Europe and North America.^2,3^ Although GBS can occur in any age group, its incidence increases with age.^2,3,6^ Sejvar et al^2^ reported that the incidence of GBS is 0.62 cases per 100 000 person-years in children, 1.85 cases per 100 000 person-years among adults aged 60 to 69 years, and 2.66 cases per 100 000 person-years among adults aged ≥80 years.

Despite its rarity, GBS is an important topic of discussion when vaccinating against influenza. A landmark study conducted during the swine flu outbreak in the United States in 1976 found that the influenza vaccine increased the risk of GBS by up to 8-fold.^7^ The risk was at its highest 2 to 3 weeks after vaccination, with an elevated risk lasting to 42 days.^7,8,9,10^ Given the general administration of the influenza vaccine among older adults and the higher prevalence of GBS among this age group, the safety of influenza vaccination in this population merits attention. Studies published since the release of the aforementioned report have continued the debate about whether influenza vaccination increases the risk of GBS. Some studies^11,12,13,14^ have reported an association of influenza vaccination with a significantly increased risk of GBS, whereas other studies have not.^15,16,17,18,19,20,21^

Because of the rarity of GBS, the discrepancies among current findings cannot be reconciled. Influenza vaccination is recommended not only for older individuals but also for younger individuals with specific conditions, such as those who are immunocompromised. Comparisons of the incidence of GBS between vaccinated and unvaccinated individuals in cohort or case-control studies have been reported previously.^17,22^ Moreover, the incidence of GBS may increase falsely after vaccination if cases are intentionally reported to adverse drug event registries. Thus, identifying an appropriate control population for comparison is difficult owing to a global vaccination campaign targeted at the general older population.

Self-controlled case series (SCCS)^23,24^—which are based on a case-only approach that automatically controls for individual-level, time-invariant confounders—have gained recognition as a reliable method for examining the association between vaccination and adverse effects.^25,26^ More than 99% of the population of Taiwan (ie, 23 million residents) is covered by the National Health Insurance (NHI), a universal health care program launched in 1995.^27^ The policy of free annual influenza vaccination for all individuals 65 years or older was implemented by Taiwan’s Centers for Disease Control through the NHI system in 2001. Each year, enrolled older individuals can visit any NHI-licensed clinic or hospital to receive free influenza vaccination.^28^ Thus, the use of Taiwan’s NHI Research Database (NHIRD) with the SCCS method may provide a unique advantage in examining the association between influenza vaccination and GBS.

If influenza vaccination is not associated with GBS, the onset of GBS in selected patients would likely be distributed equally over the entire observation period before and after vaccination. We conducted a nationwide study of adults aged 65 years or older in Taiwan to investigate the risk of GBS after seasonal influenza vaccination among this population.

## Methods

In this population-based, retrospective cross-sectional study using the SCCS method, data were retrieved between January 1, 2003, and December 31, 2017. from Taiwan’s NHIRD published by the Health and Welfare Data Science Center, Ministry of Health and Welfare. The study protocol was approved by the Taichung Jen-Ai Hospital Institutional Review Board; informed consent was waived owing to anonymous data that were retrieved retrospectively. This study followed the Strengthening the Reporting of Observational Studies in Epidemiology (STROBE) reporting guideline.

### Data Source

We conducted a secondary data analysis using data from Taiwan’s NHIRD covering the period 2002 to 2018. The NHIRD is a population-based health database that includes the details of beneficiaries enrolled in Taiwan’s NHI. Information provided in the NHIRD, including detailed clinical records on outpatient visits, hospitalizations, diagnostic codes, and prescriptions, is highly concordant with NHI claims records and patient self-reports.^29^

### Study Design

**Figure 1.**
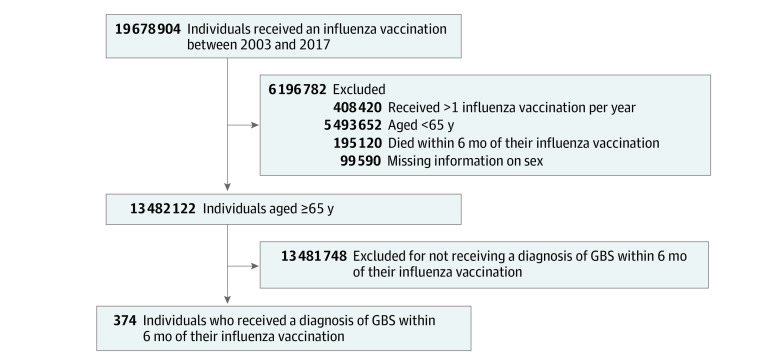
Study Flowchart GBS indicates Guillain-Barré syndrome.

Adults aged 65 years or older who had received influenza vaccination and were hospitalized for GBS between January 1, 2003, and December 31, 2017, were enrolled in the study. These individuals were selected because this age group benefits from Taiwan’s free annual influenza vaccination policy. Using the NHIRD data, we identified inpatients with GBS based on *International Classification of Diseases, Ninth Revision, Clinical Modification* code 357.0 and *International Classification of Diseases, Tenth Revision, Clinical Modification* code G61.0. After excluding patients who (1) received influenza vaccination more than once per year, (2) died within 6 months after receiving their influenza vaccination, and (3) had missing values for any study variables, we included 374 older patients who received a diagnosis of GBS within 6 months of their influenza vaccination (Figure 1). Control variables in this study included sex, age, Charlson Comorbidity Index (CCI) severity,^30^ and comorbidities. Age groups were categorized as ages 65 to 74 years, 75 to 84 years, and 85 years or older. The CCI scores were categorized as lower than 2 and 2 or higher, the latter of which indicated that the patient had at least 2 chronic comborbid diseases and/or a medical condition that posed moderate to severe risks to the patient’s health. Comorbidities included cancer and autoimmune diseases, which were identified according to NHIRD data in the registry for catastrophic illness under the NHI, and were categorized as yes or no.

### Definition of GBS Onset

**Figure 2.**
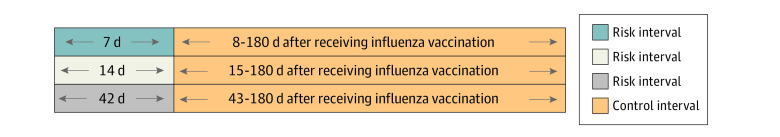
Risk Intervals and Control Intervals

The date of GBS onset was defined as the admission date with GBS coded during the study period. The follow-up period was 180 days from the date of vaccination. According to reports,^7,8,9,10^ the possible increased risks for developing GBS persist for up to 6 weeks after vaccination and are highest in the first 2 to 3 weeks. Given the available background information, we defined the risk period as 3 intervals comprising the first 7, 14, and 42 days after influenza vaccination; the corresponding control periods were defined as days 8 to 180, days 15 to 180, and days 43 to 180, respectively (Figure 2). If GBS was not related to influenza vaccination, the incidence of GBS in the study patients would presumably be distributed equally during the entire observation period.

### Statistical Analysis

Data were analyzed from November 1, 2021, through February 28, 2022. We performed Poisson regression to analyze the incidence rate ratio (IRR) and 95% CI for incident GBS during the risk and control intervals. The model accounted for hospitalizations for GBS per patient during the study period. In addition, we evaluated the risk during the 3 intervals that comprised the risk period (ie, days 1-7, days 1-14, and days 1-42). Additionally, we performed a stratified analysis according to age group (65-74 years, 75-84 years, and ≥85 years), sex, CCI (score <2 and ≥2), autoimmune disease, and cancer. All statistical analyses were performed using SAS, version 9.4 (SAS Institute Inc); 2-sided *P* < .05 was considered statistically significant.

## Results

A total of 13 482 122 adults aged 65 years or older received an influenza vaccination; 374 of these adults were hospitalized for GBS and thus comprised the study population. The mean (SD) age was 75.0 (6.1) years; 215 individuals (57.5%) were men and 159 (42.5%) were women. One hundred eighty three individuals (48.9%) were aged 65 to 74 years, 147 (39.3%) were aged 75 to 84 years, and 44 (11.8%) were 85 years or older. In terms of comorbidities, 33 individuals (8.8%) had cancer and 4 (1.1%) had autoimmune disease. Baseline characteristics of the study population are provided in Table 1.

**Table 1.** Baseline Characteristics of the Study Population^a^

Characteristic	Values
Total No. of individuals	374 (100)
Age, mean (SD), y	75.0 (6.1)
Age group, y	
65-74	183 (48.9)
75-84	147 (39.3)
≥85	44 (11.8)
Sex	
Male	215 (57.5)
Female	159 (42.5)
Charlson Comorbidity Index	
<2	140 (37.4)
≥2^b^	234 (62.6)
Cancer	
No	341 (91.2)
Yes	33 (8.8)
Autoimmune disease	
No	370 (98.9)
Yes	4 (1.1)

^a^ Unless indicated otherwise, data are presented as No. (%) of individuals.

^b^ Indicated that the patient had at least 2 chronic comborbid diseases and/or a medical condition that posed moderate to severe risks to the patient’s health.

Table 2 presents details regarding the risk of GBS after influenza vaccination. The IRR for days 1 to 42 (ie, the risk period) compared with days 43 to 180 (ie, the overall control period) was 0.92 (95% CI, 0.72-1.17; *P* = .49). We observed no significant increase in GBS incidence during risk intervals for days 1 to 7 (IRR, 0.95 [95% CI, 0.55-1.61; *P* = .84]) or days 1 to 14 (IRR, 0.87 [95% CI, 0.58-1.29; *P* = .48]).

**Table 2.** Incidence Rate Ratio (IRR) of Guillain-Barré Syndrome After Influenza Vaccination^a^

Risk interval	IRR (95% CI)	*P* value
Days 1-7 (vs days 8-180)	0.95 (0.55-1.61)	.84
Days 1-14 (vs days 15-180)	0.87 (0.58-1.29)	.48
Days 1-42 (vs days 43-180)	0.92 (0.72-1.17)	.49

^a^ Estimated using Poisson regression after adjustment for baseline characteristics (Table 1).

The results of subgroup analyses are provided in Table 3. The IRRs on days 1 to 42 among individuals aged 65 to 74 years, 75 to 84 years, and 85 years or older were 0.93 (95% CI, 0.66-1.31), 0.85 (95% CI, 0.58-1.26), and 1.10 (95% CI, 0.57-2.11), respectively. Similarly, we observed no increase in GBS incidence across the 3 age groups on days 1 to 7 (IRR, 1.17 [95% CI, 0.60-2.29; *P* = .64]; IRR, 0.48 [95% CI, 0.15-1.51; *P* = .21]; IRR, 1.11 [95% CI, 0.27-4.58; *P* = .89], respectively) or days 1 to 14 (IRR, 1.02 [95% CI, 0.60-1.73; *P* = .94]; IRR, 0.67 [95% CI, 0.33-1.37; *P* = 28]; IRR, 0.87 [95% CI, 0.27-2.80; *P* = .81], respectively). In subgroup analyses by sex, the IRR did not increase significantly on days 1 to 7 (men, 0.99 [95% CI, 0.51-1.94; *P* = .98]; women, 0.75 [95% CI, 0.31-1.83; *P* = .53]), days 1 to 14 (men, 0.86 [95% CI, 0.51-1.46; *P* = .58]; women, 0.87 [95% CI, 0.47-1.60; *P* = .66]), or days 1 to 42 (men, 0.97 [95% CI, 0.71-1.33; *P* = .87]; women, 0.85 [95% CI, 0.58-1.23; *P* = .39]). The results of subgroup analysis for CCI did not reach statistical significance even though the IRRs among the high-CCI (≥2) group were all greater than 1 (days 1-7, 1.12 [95% CI, 0.61-2.05; *P* = .72]; days 1-14, 1.01 [95% CI, 0.64-1.62; *P* = .95]; days 1-42, 1.03 [95% CI, 0.77-1.38; *P* = .84]) and those among the low-CCI (<2) group were all less than 1 (days 1-7, 0.51 [95% CI, 0.16-1.60; *P* = .25]; days 1-14, 0.62 [95% CI, 0.29-1.32; *P* = .22]; days 1-42, 0.75 [95% CI, 0.49-1.13; *P* = .17]).

**Table 3.** Comparison of Guillain-Barré Syndrome Incidence Rate Ratios (IRRs) After Influenza Vaccination by Risk Interval^a^

Characteristic	Risk interval					
	Days 1-7		Days 1-14		Days 1-42	
	IRR (95% CI)	*P* value	IRR (95% CI)	*P* value	IRR (95% CI)	*P* value
Age group, y						
65-74	1.17 (0.60-2.29)	.64	1.02 (0.60-1.73)	.94	0.93 (0.66-1.31)	.68
75-84	0.48 (0.15-1.51)	.21	0.67 (0.33-1.37)	.28	0.85 (0.58-1.26)	.43
≥85	1.11 (0.27-4.58)	.89	0.87 (0.27-2.80)	.81	1.10 (0.57-2.11)	.79
Sex						
Male	0.99 (0.51-1.94)	.98	0.86 (0.51-1.46)	.58	0.97 (0.71-1.33)	.87
Female	0.75 (0.31-1.83)	.53	0.87 (0.47-1.60)	.66	0.85 (0.58-1.23)	.39
Charlson Comorbidity Index						
<2	0.51 (0.16-1.60)	.25	0.62 (0.29-1.32)	.22	0.75 (0.49-1.13)	.17
≥2^b^	1.12 (0.61-2.05)	.72	1.01 (0.64-1.62)	.95	1.03 (0.77-1.38)	.84
Cancer						
No	0.91 (0.52-1.58)	.74	0.84 (0.55-1.28)	.42	0.94 (0.74-1.21)	.65
Yes	0.71 (0.10-5.16)	.73	1.15 (0.35-3.75)	.82	0.68 (0.28-1.64)	.39
Autoimmune disease						
No	0.90 (0.53-1.54)	.70	0.88 (0.59-1.30)	.51	0.92 (0.72-1.17)	.48
Yes^c^	NA	NA	NA	NA	1.10 (0.11-10.53)	.94

Abbreviation: NA, not applicable.

^a^ Estimated using Poisson regression after adjustment for baseline characteristics (Table 1) but without including the stratified variable.

^b^ Indicated that the patient had at least 2 chronic comborbid diseases and/or a medical condition that posed moderate to severe risks to the patient’s health.

^c^ Data are not reported for days 1 to 7 or days 1 to 14 owing to the small sample size.

We observed no increased risk of GBS after influenza vaccination among patients with cancer or autoimmune disease as comorbidities. Among patients with cancer, the IRRs on days 1 to 7, days 1 to 14, and days 1 to 42 were 0.71 (95% CI, 0.10-5.16; *P* = .73), 1.15 (95% CI, 0.35-3.75; *P* = .82), and 0.68 (95% CI, 0.28-1.64; *P* = .39), respectively. Among patients with autoimmune disease, the IRR on days 1 to 42 was 1.10 (95% CI, 0.11-10.53; *P* = .94) (data for days 1-7 and days 1-14 are not reported owing to the small sample size).

## Discussion

We found no significantly increased risk of GBS among adults 65 years or older in Taiwan during the first 42 days after influenza vaccination. Subgroup analyses by sex and age yielded consistent results. To our knowledge, this study includes the largest number of older adults who developed GBS after influenza vaccination and thus has more strength of evidence among populations of Asian individuals. Older adults have a higher risk of complications from influenza infection and therefore stand to benefit the most from influenza vaccination. Our findings suggest that the benefit of the influenza vaccine may outweigh the potential concern of GBS risk in this population.

The definition of risk periods merits further discussion. In previous studies, GBS risk was reported to last for up to 6 weeks after vaccination and was at its highest 2 to 3 weeks after vaccination.^7,8,9,10^ Thus, most studies examining the association between GBS and influenza vaccines have defined the risk period as 42 days after vaccination, including a recent study by Grave et al^21^ that found no association between seasonal influenza vaccination and GBS among a large cohort in France. However, Haber et al^9^ reported that most patients (59%) with GBS exhibited symptoms within 14 days of vaccination. To identify the periods of the highest risk for GBS within 6 weeks, we investigated the risk intervals that comprised days 1 to 7 and 1 to 14 and did not observe an association between influenza vaccination and GBS during these periods.

GBS is usually associated with prior respiratory or gastrointestinal infections; however, it has also been reported in patients with comorbid malignancies.^31,32,33^ Cancer may increase the risk of GBS by disrupting the immune system,^33^ but the link between cancer and GBS remains under debate. Additionally, GBS has been reported to occur concomitantly with several connective tissue diseases.^34,35,36^ Using an SCCS-based study design, we found that IRRs for GBS did not increase or decrease significantly among individuals with comorbid cancer (0.68 [95% CI, 0.28-1.64]) or autoimmune disease (1.10 [95% CI, 0.11-10.53]) after influenza vaccination. As a point of interest, the SCCS approach can be used to investigate a possible temporal link between influenza vaccination and GBS; however, this method cannot compare outcomes among individuals with and without specific medical conditions such as cancer or autoimmune disease. Future research should incorporate alternative research designs such as case-control studies or randomized clinical trials to explore the comparative differences between subgroups.

### Limitations

This study has several limitations. First, antigens in influenza vaccines change each year, and even batches of a particular vaccine can differ. All influenza vaccines used in Taiwan from 2003 to 2017 were denatured virus-based vaccines; therefore, we did not divide the different brands of influenza vaccines into categories. Second, we included only individuals who had been hospitalized for GBS. Patients with GBS who did not require hospitalization owing to mild symptoms were not analyzed, and those who died were not included. Thus, the number of GBS cases after vaccination is likely to be underestimated; however, the same is true for cases that are unrelated to vaccination. Third, because of the nature of GBS, determining the onset date was difficult. The date of disease onset used in this study may have been inaccurate, and the lag (or latent period) was due to patients not having been hospitalized at the onset of their clinical symptoms. Because an error of a few days might exist in some cases, we measured the risk period in weeks; therefore, it is unlikely that the discrepancy substantially affected the outcome. Fourth, the SCCS method controls all time-invariant confounders during the study period. However, potential time-varying confounders such as seasonality and respiratory tract and gastrointestinal infections were not controlled. Our results should therefore be interpreted with caution.

## Conclusions

The findings of this population-based, cross-sectional study with an SCCS design suggest that there was no increase in the risk of GBS after influenza vaccination among adults older than 65 years regardless of postvaccination period. Future research that incorporates alternate study designs (eg, case-control studies or randomized clinical trials) are needed to confirm our findings.
